# Supplementary material for: AI-based antibody design targeting recent H5N1 avian influenza strains
Source: Comput Struct Biotechnol J. 2025 Jun 27;27:2915–23. doi: 10.1016/j.csbj.2025.06.026 (PMC12270607; doi:10.1016/j.csbj.2025.06.026)
Supplement: MMC — Sequence identity comparisons between the diffused and reference antibody sequences. A) Violin/box plots showing the distribution of the diffused sequences' identities by chain. B) Heatmap showing the pairwise identity comparisons. [file mmc1.pdf]

Supplementary Materials

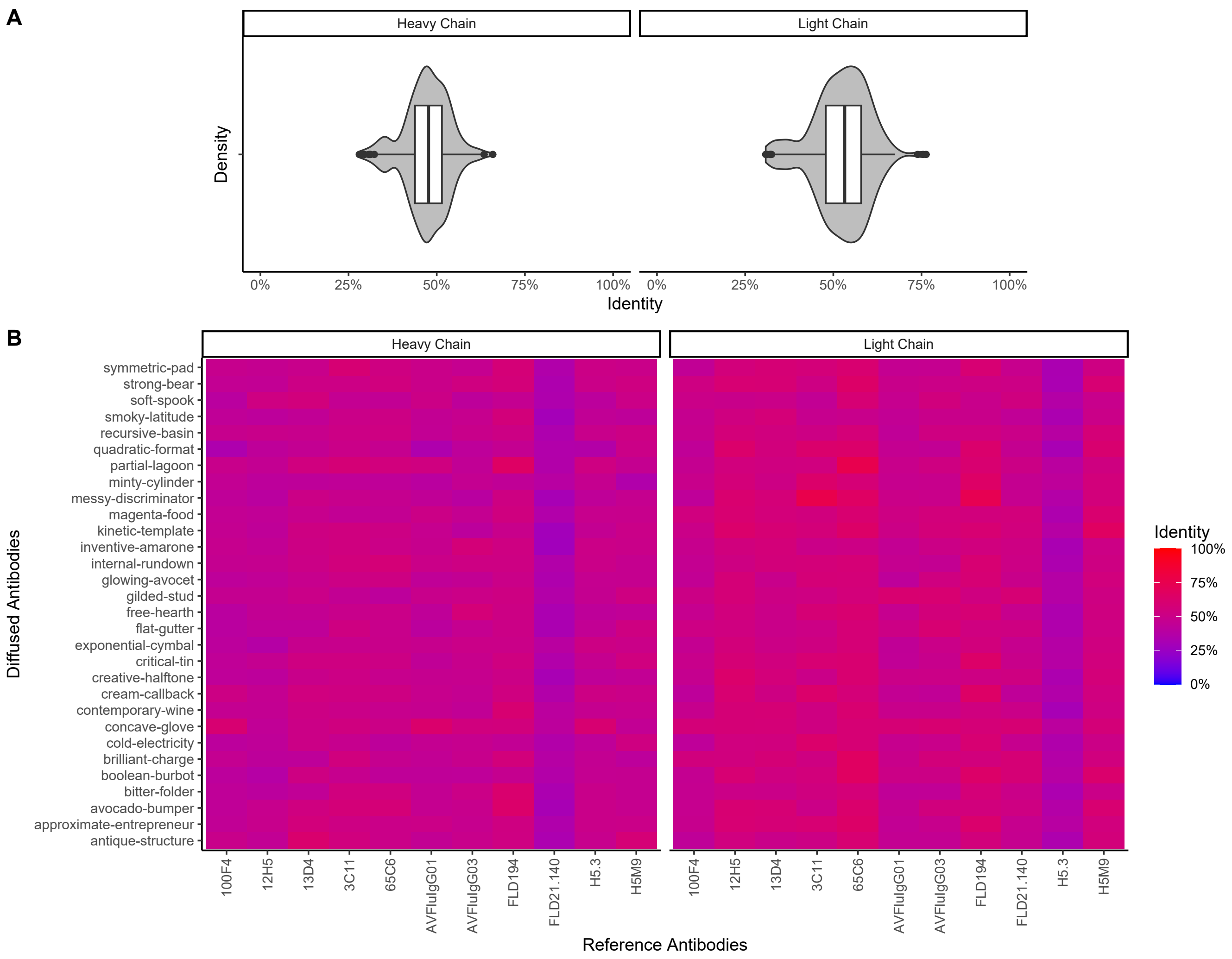

Fig. 1. Sequence identity comparisons between the diffused and reference antibody sequences. A) Violin/box plots showing the distribution of the diffused sequences' identities by chain. B) Heatmap showing the pairwise identity comparisons.
